# Comparison of the crystal structures of the low- and high-temperature forms of bis­[4-(di­methyl­amino)­pyridine]­dithio­cyanato­cobalt(II)

**DOI:** 10.1107/S2056989021010422

**Published:** 2021-10-19

**Authors:** Christoph Krebs, Inke Jess, Christian Näther

**Affiliations:** a Institute of Inorganic Chemistry, University of Kiel, Max-Eyth.-Str. 2, 24118 Kiel, Germany

**Keywords:** crystal structure, polymorphism, cobalt(II)thio­cyanate, hydrogen bonding

## Abstract

Single crystals of the high-temperature form I of [Co(NCS)_2_(DMAP)_2_] (DMAP = 4-di­methyl­amino­pyridine, C_7_H_10_N_2_) were obtained accidentally by the reaction of Co(NCS)_2_ with DMAP at slightly elevated temperatures under kinetic control. This modification crystallizes in the monoclinic space group *P*2_1_/*m* and is isotypic with the corresponding Zn compound. The asymmetric unit consists of one crystallographically independent Co cation and two crystallographically independent thio­cyanate anions that are located on a crystallographic mirror plane and one DMAP ligand (general position). In its crystal structure the discrete complexes are linked by C—H⋯S hydrogen bonds into a three-dimensional network. For comparison, the crystal structure of the known low-temperature form II, which is already thermodynamically stable at room temperature, was redetermined at the same temperature. In this polymorph the complexes are connected by C—H⋯S and C—H⋯N hydrogen bonds into a three-dimensional network. At 100 K the density of the high-temperature form I (ρ = 1.462 g cm^−3^) is higher than that of the low-temperature form II (ρ = 1.457 g cm^−3^), which is in contrast to the values determined by XRPD at room temperature. Therefore, these two forms represent an exception to the Kitaigorodskii density rule, for which extensive inter­molecular hydrogen bonding in form II might be responsible.

## Abstract 2

The crystal structures of the low-temperature and the already published high-temperature forms of Co(NCS)_2_(DMAP)_2_ (DMAP = 4-di­methyl­amino­pyridine) were determined at 100 K, indicating that these two forms represent an exception from the Kitaigorodskii density rule.

## Chemical context

Polymorphism and isomerism is a widespread phenomenon in coordination chemistry (Braga & Grepioni, 2000 ▸; Moulton & Zaworotko, 2001 ▸; Batten *et al.*, 1998 ▸; Zhang *et al.*, 2009 ▸). On one hand, these phenomena are a disadvantage for rational crystal design, but on the other hand they are of advantage for studying structure–property relationships (Braga *et al.*, 2001 ▸; Tao *et al.*, 2012 ▸; Ossinger *et al.*, 2020 ▸; Sheu *et al.*, 2009 ▸). Because in such a case the composition of the different forms is identical, all changes in the physical properties can be directly correlated with the structural changes. One class of compounds in which polymorphism and especially isomerism is observed are coordination compounds based on transition-metal thio­cyanates, because this anionic ligand shows several different coordination modes leading to a large structural variability (Böhme *et al.*, 2020 ▸; Jochim *et al.*, 2020 ▸; Mautner *et al.*, 2018 ▸; Neumann *et al.*, 2020*a* ▸; Wellm *et al.*, 2020*a* ▸; Werner *et al.*, 2015 ▸; Buckingham, 1994 ▸; Barnett *et al.*, 2002 ▸).

In this context, we have recently reported the crystal structure of form II of Co(NCS)_2_(DMAP)_2_ (DMAP = 4-di­methyl­amino­pyridine, C_7_H_10_N_2_), which crystallizes as discrete complexes in which the cobalt cations are tetra­hedrally coordinated. This modification can directly be obtained from the reaction of Co(NCS)_2_ and 4-di­methyl­amino­pyridine in aqueous solution or by thermal decomposition of Co(NCS)_2_(DMAP)_2_(H_2_O)_2_-dihydrate (Neumann *et al.*, 2018*a* ▸). In contrast, if the methanol complex Co(NCS)_2_(DMAP)_2_(MeOH)_2_ is thermally decomposed, a new polymorphic modification of Co(NCS)_2_(DMAP)_2_ (form I) is obtained. Because we were not able to prepare single crystals of this form, the corresponding Zn complex was prepared and XRPD indicates that it is isotypic to form I of the Co compound (Neumann *et al.*, 2018*b* ▸). Solvent-mediated conversion experiments reveal that form II is the thermodynamically stable form at room temperature and transforms into form I upon heating. Both forms are related by enanti­otropism and the thermodynamic transition temperature was determined to be above 135°C. The metastability of form I at room temperature might be the reason why no single crystals were obtained. It is noted that in contrast to the Co modification I, the corresponding Zn form is already thermodynamically stable at room temperature, which might be the reason that single crystals of this form can easily be prepared from solution (Neumann *et al.*, 2020*a* ▸,*b* ▸). 
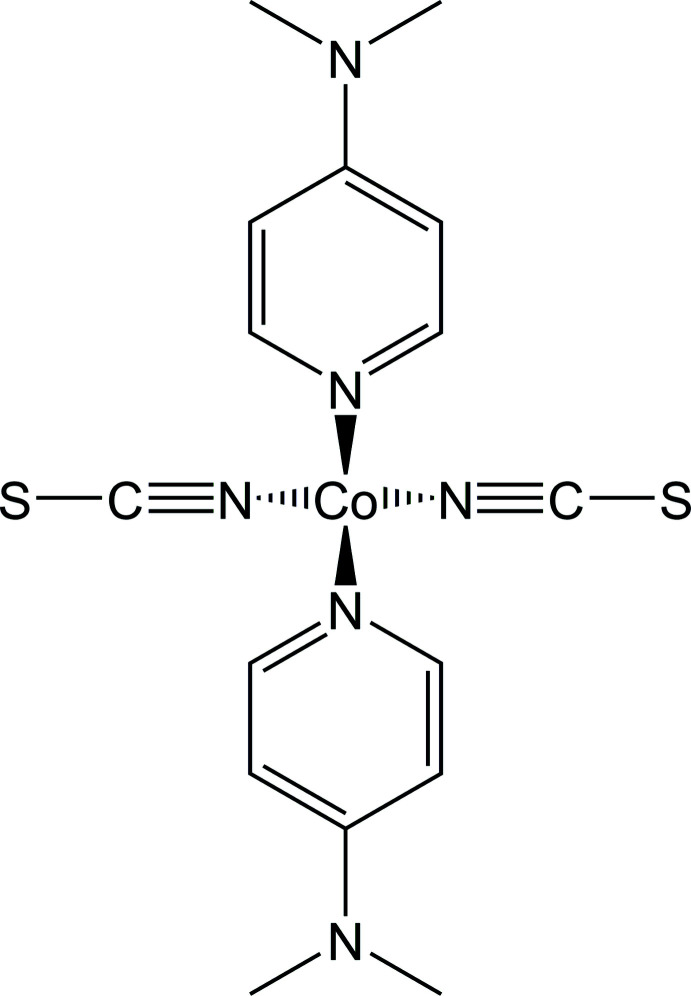


Later on, we investigated whether the physical properties of thio­cyanate coordination compounds can be influenced by mixed crystal formation and we found out that, for example, the critical temperature in layered thio­cyanate networks can be tuned by preparing mixed crystals with Co(NCS)_2_ and Ni(NCS)_2_ where a linear increase of *T*_c_ with increasing Co content was observed (Neumann *et al.*, 2018 **b* ▸;* Wellm *et al.*, 2018 ▸, 2020*b* ▸). In the course of our systematic work, we are currently investigating whether mixed crystals of Ni(NCS)_2_(DMAP)_2_ and Co(NCS)_2_(DMAP)_2_ can be prepared. As already noted, the Co compound forms discrete complexes whereas the Ni compound shows a chain structure (Jochim *et al.*, 2018 ▸). Preliminary XRPD investigations indicate that, in those cases where more than 50% Co(NCS)_2_ is used in the synthesis, a very small amount of form I of Co(NCS)_2_(DMAP)_2_ is formed as a side phase. This is in agreement with crystallization experiments to obtain single crystals where Co(NCS)_2_ and Ni(NCS)_2_ were used in a 90:10 ratio, because block-shaped and needle-like crystal are visible. Both of them were identified by single crystal X-ray diffraction, which proves that the block-like crystals correspond to the unit cell of the Ni compound, whereas the needle-like crystals correspond to the metastable form I of Co(NCS)_2_(DMAP)_2_, which was obviously obtained accidentally under kinetic control. To exclude the possibility that mixed crystals of form I have formed, the crystallization reaction was repeated with only Co(NCS)_2_ and in this case the same crystalline phase was obtained. As mentioned above, its single-crystal structure is unknown and it is therefore presented here for the first time. For better comparison, we also present the structure of form II at 100 K, because in our previous work it was measured at 170 K (Neumann *et al.*, 2018*b* ▸).

## Structural commentary

**Figure 1:**
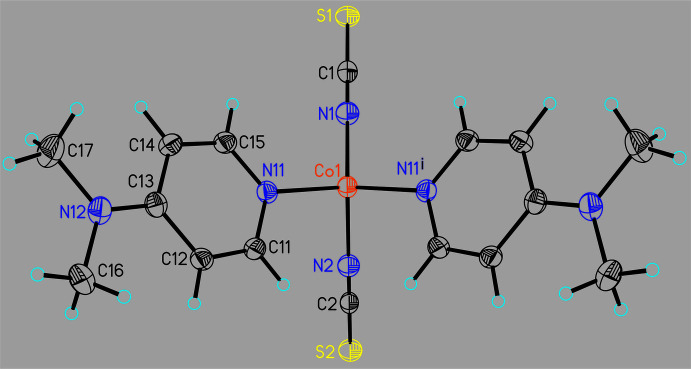
Crystal structure of form I with labeling and displacement ellipsoids drawn at the 50% probability level. Symmetry code: (i) = *x*, 3/2 − y, *z*.

Form I of Co(NCS)_2_(DMAP)_2_ crystallizes in the monoclinic space group *P*2_1_/*m* with *Z* = 2 and the Co cation as well as the thio­cyanate anions are located on a crystallographic mirror plane, whereas the known form II crystallizes in space group *P*2_1_/*c* with *Z* = 4 with all atoms in general positions. In both modifications, the Co^II^ cations are fourfold coordinated by two terminal N-bonded thio­cyanate anions and two DMAP ligands within slightly distorted tetra­hedral environments (Figs. 1 ▸ and 2 ▸ and Table 1 ▸). In form I, the two Co—N bond lengths to the thio­cyanate anions are slightly different, which is not the case in form II (Table 2 ▸). Usually this is reflected in the values of the CN stretching vibrations but this is not the case for form I, because two bands are expected but only one is visible in its IR spectrum (Neumann *et al.*, 2018*b* ▸). Moreover, the Co—N bond lengths to the DMAP ligands are slightly longer in form I compared to form II (Table 2 ▸). From the N—Co—N bond angles, it is obvious that both tetra­hedra are slightly distorted (Table 1 ▸). In both modifications, the Co—N—C bond angle is close to linear. Finally, it is noted that the density of form I at 100 K of 1.462 g cm^−3^ is significantly greater than that of form II (1.457 g cm^−3^). This is surprising because form I was proven to be thermodynamically stable at a lower temperature and should have the higher density according to the density rule (Kitaigorodskii, 1961 ▸). This was determined from a Pawley fit of a powder pattern measured at room temperature (Neumann *et al.*, 2018*b* ▸) and therefore, the current findings are somehow in contradiction to the previous findings. Other exceptions to this rule are known if the crystal structure is dominated by inter­molecular hydrogen bonding, as already discussed in the literature (Burger & Ramberger, 1979 ▸).

## Supra­molecular features

**Figure 3:**
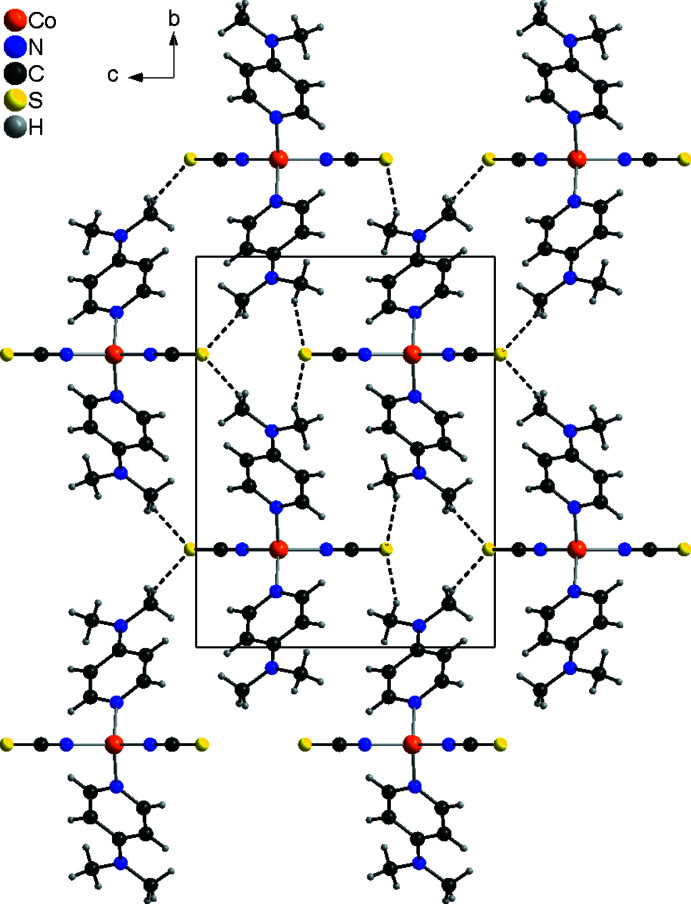
Crystal structure of form I with a view of a layer in the direction of the crystallographic *a-*axis. Inter­molecular C—H⋯S hydrogen bonding is shown as dashed lines.

**Figure 4:**
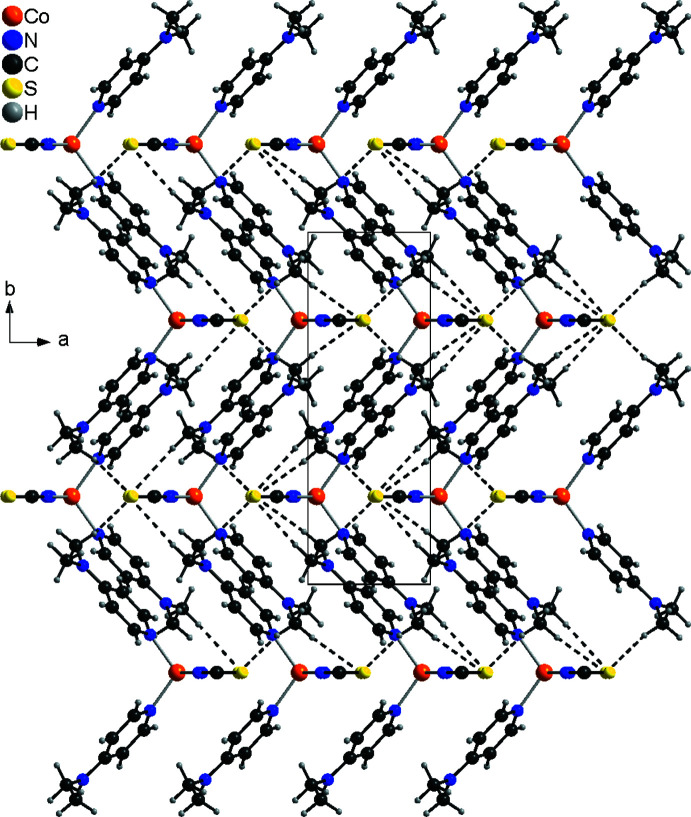
Crystal structure of form I viewed in the direction of the crystallographic *c*-axis. Inter­molecular C—H⋯S hydrogen bonding is shown as dashed lines.

In the crystal structure of form I, the discrete complexes are linked by C—H⋯S hydrogen bonds between one of the DMAP methyl H atoms and the thio­cyanate S atoms into layers that lie parallel to the *bc* plane (Fig. 3 ▸). In this arrangement, each of the two S atoms acts as an acceptor for two hydrogen bonds to two symmetry-equivalent DMAP ligands (Fig. 3 ▸). The C—H⋯S angles are close to 180°, indicating a relatively strong inter­action (Table 3 ▸). These layers are further connected by weaker C—H⋯S contacts involving the thio­cyanate S atom S1 and the methyl H atoms of the DMAP ligands (Fig. 4 ▸).

**Figure 5:**
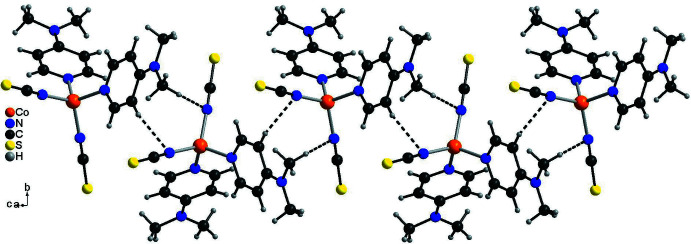
Crystal structure of form II with a view of a chain with inter­molecular C—H⋯S hydrogen bonding shown as dashed lines.

**Figure 6:**
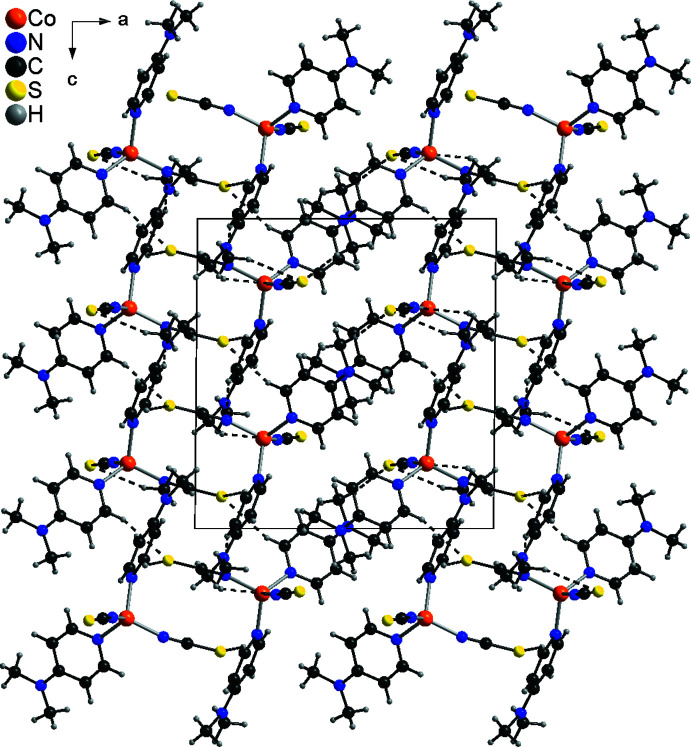
Crystal structure of form II with view in the direction of the crystallographic *b*-axis. Inter­molecular C—H⋯S and C—H⋯N hydrogen bonds are shown as dashed lines.

In contrast to form I, both hydrogen bonds, C—H⋯S and C—H⋯N, are present in form II. In this modification, the mol­ecules are linked by pairs of C—H⋯N hydrogen bonds between the thio­cyanate N atoms and the H atoms of the DMAP ligands into chains that propagate along the crystallographic *c*-axis direction (Fig. 5 ▸). These chains are further linked into a complicated three-dimensional network by four different C—H⋯S hydrogen bonds between the hydrogen atoms of the DMAP ligands and the thio­cyanate S atoms (Fig. 6 ▸ and Table 4 ▸). For three of these hydrogen bonds, the C—H⋯S angle is close to linearity, which indicates that it is a relatively strong inter­action. This extensive inter­molecular hydrogen bonding might be responsible for the fact that the density of the low-temperature form II at 100 K is lower than that of the high-temperature form I, which is an exception to the density rule.

## Database survey

As mentioned in the *Chemical context* section, the single-crystal structure of form II and the thermodynamic relations between form I and form II have already been reported (Neumann *et al.*, 2018*b* ▸). Also related are the corresponding Zn(NCS)_2_ modifications, but in contrast to Co, three different forms were observed with Zn (Neumann *et al.*, 2018*a* ▸,*b* ▸).

However, compounds with DMAP and other transition-metal thio­cyanates also exist. This includes the compound Zn(NCS)_2_(DMAP)_2_·chloro­benzene (Cambridge Structural Database refcode: QIPXES; Secondo *et al.*, 2000 ▸), where the metal center is tetra­hedrally coordinated. In addition, some octa­hedral complexes are known in the literature. Cu(NCS)_2_(DMAP)_2_(di­methyl­formamide)_2_ (HIVZAO; Chen *et al.*, 2007 ▸), Mn(NCS)_2_(DMAP)_2_(CH_3_OH)_2_ (NUKCON; Suckert *et al.*, 2015 ▸) and Cd(NCS)_2_(DMAP)_2_(DMSO)_2_ (QIPXOC; Secondo *et al.*, 2000 ▸) all consist of a metal center with two thio­cyanate anions, two DMAP co-ligands and two additional identical co-ligands each.

In [Cd(NCS)_2_(DMAP)_2_]_*n*_ (QIPXIW; Secondo *et al.*, 2000 ▸) and [Ni(NCS)_2_(DMAP)_2_]_*n*_ (GIQQOP; Jochim *et al.*, 2018 ▸), two non-isotypical linear chains are reported, in which the cations have an all-*trans M*N_4_S_2_ octa­hedral coordination of two N-bonded and two S-bonded bridging thio­cyanate anions and two DMAP co-ligands.

## Synthesis and crystallization

Co(NCS)_2_ and DMAP were purchased from Merck. All chemicals were used without further purification.

Blue single crystals of form I suitable for single crystal X-ray analysis were obtained three days after storing 0.15 mmol Co(NCS)_2_ (26.3 mg) and 0.30 mmol DMAP (36.6 mg) in 1.0 ml H_2_O at 333 K followed by slow cooling.

Single crystals of form II were obtained as described in the literature (Neumann *et al.*, 2018*a* ▸).

## Refinement

The C-bound H atoms were located in the difference map but positioned with idealized geometry (C—H = 0.95–0.98 Å; methyl H atoms allowed to rotate but not to tip) and were refined isotropically with *U*_iso_(H) = 1.2*U*_eq_(C) [1.5*U*_eq_(C) for methyl H atoms] using a riding model. Crystal data, data collection and structure refinement details are summarized in Table 5 ▸.

## Supplementary Material

Crystal structure: contains datablock(s) Form_I, Form_II. DOI: 10.1107/S2056989021010422/hb7987sup1.cif

Structure factors: contains datablock(s) Form_I. DOI: 10.1107/S2056989021010422/hb7987Form_Isup2.hkl

Structure factors: contains datablock(s) Form_II. DOI: 10.1107/S2056989021010422/hb7987Form_IIsup3.hkl

CCDC references: 2114553, 2114552

Additional supporting information:  crystallographic information; 3D view; checkCIF report

## Figures and Tables

**Figure 2:**
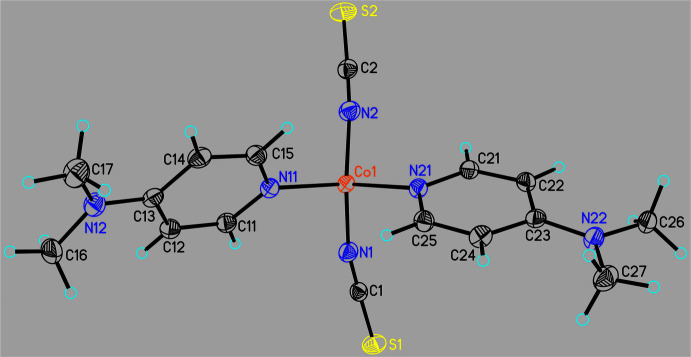
Crystal structure of form II with labeling and displacement ellipsoids drawn at the 50% probability level.

**Table 1:** Selected geometric parameters (Å, °) for form I[Chem scheme1]

Co1—N1	1.9429 (18)	Co1—N11	2.0148 (12)
Co1—N2	1.9672 (19)		
			
N1—Co1—N2	118.16 (8)	N11—Co1—N11^i^	109.04 (7)
N1—Co1—N11	111.03 (4)	C1—N1—Co1	179.48 (17)
N2—Co1—N11	103.47 (4)	C2—N2—Co1	166.24 (16)

Symmetry code: (i) x, -y+{\script{3\over 2}}, z.

**Table 2:** Selected geometric parameters (Å, °) for form II[Chem scheme1]

Co1—N1	1.9521 (13)	Co1—N11	2.0057 (12)
Co1—N2	1.9535 (14)	Co1—N21	2.0013 (12)
			
N1—Co1—N2	117.81 (6)	N2—Co1—N21	106.71 (5)
N1—Co1—N11	105.83 (5)	N21—Co1—N11	112.36 (5)
N1—Co1—N21	105.41 (5)	C1—N1—Co1	168.56 (12)
N2—Co1—N11	108.82 (5)	C2—N2—Co1	175.34 (13)

**Table 3:** Hydrogen-bond geometry (Å, °) for form I[Chem scheme1]

*D*—H⋯*A*	*D*—H	H⋯*A*	*D*⋯*A*	*D*—H⋯*A*
C16—H16*B*⋯S2^ii^	0.98	2.91	3.8367 (15)	158
C17—H17*B*⋯S1^iii^	0.98	2.94	3.7291 (16)	138
C17—H17*C*⋯S1^iv^	0.98	2.89	3.8018 (18)	155

Symmetry codes: (ii) -x+1, -y+1, -z+1; (iii) -x+1, -y+1, -z; (iv) -x+2, -y+1, -z.

**Table 4:** Hydrogen-bond geometry (Å, °) for form II[Chem scheme1]

*D*—H⋯*A*	*D*—H	H⋯*A*	*D*⋯*A*	*D*—H⋯*A*
C11—H11⋯S1^i^	0.95	2.87	3.7312 (15)	152
C16—H16*C*⋯S2^ii^	0.98	3.01	3.9663 (16)	166
C21—H21⋯S1^iii^	0.95	2.92	3.7888 (15)	153
C22—H22⋯N1^iv^	0.95	2.64	3.5448 (19)	159
C26—H26*A*⋯N2^iv^	0.98	2.70	3.531 (2)	143
C27—H27*B*⋯S2^iv^	0.98	2.98	3.7568 (17)	137

Symmetry codes: (i) -x+2, -y+1, -z+2; (ii) -x+1, -y+1, -z+2; (iii) -x+2, y-{\script{1\over 2}}, -z+{\script{3\over 2}}; (iv) x, -y+{\script{1\over 2}}, z-{\script{1\over 2}}.

**Table 5:** Experimental details

	Form I	Form II
Crystal data		
Chemical formula	[Co(NCS)_2_(C_7_H_10_N_2_)_2_]	C_16_H_20_CoN_6_S_2_
*M*_r_	419.43	419.43
Crystal system, space group	Monoclinic, *P*12_1_/*m*1	Monoclinic, *P*2_1_/*c*
Temperature (K)	100	100
*a*, *b*, *c* (Å)	5.3708 (1), 15.2200 (2), 11.8014 (1)	13.9171 (1), 9.5114 (1), 14.4487 (1)
β (°)	99.076 (1)	90.489 (1)
*V* (Å^3^)	952.61 (2)	1912.52 (3)
*Z*	2	4
Radiation type	Cu *K*α	Cu *K*α
μ (mm^−1^)	9.20	9.17
Crystal size (mm)	0.2 × 0.12 × 0.04	0.18 × 0.1 × 0.03

Data collection		
Diffractometer	XtaLAB Synergy, Dualflex, HyPix	XtaLAB Synergy, Dualflex, HyPix
Absorption correction	Multi-scan (*CrysAlis PRO*; Rigaku OD, 2021 ▸)	Multi-scan (*CrysAlis PRO*; Rigaku OD, 2021 ▸)
*T*_min_, *T*_max_	0.311, 1.000	0.476, 1.000
No. of measured, independent and observed [*I* > 2σ(*I*)] reflections	16411, 2100, 2081	57319, 4151, 4079
*R*_int_	0.025	0.025
(sin θ/λ)_max_ (Å^−1^)	0.635	0.639

Refinement		
*R*[*F* ^2^ > 2σ(*F* ^2^)], *wR*(*F* ^2^), *S*	0.024, 0.067, 1.14	0.026, 0.073, 1.13
No. of reflections	2100	4151
No. of parameters	126	231
H-atom treatment	H-atom parameters constrained	H-atom parameters constrained
Δρ_max_, Δρ_min_ (e Å^−3^)	0.25, −0.37	0.29, −0.35

Computer programs: *CrysAlis PRO* (Rigaku OD, 2021 ▸), *SHELXT2014/5* (Sheldrick, 2015*a* ▸), *SHELXL2016/6* (Sheldrick, 2015*b* ▸), *DIAMOND* (Brandenburg & Putz, 1999 ▸) and *publCIF* (Westrip, 2010 ▸).

## supplementary crystallographic information

### Bis[4-(dimethylamino)pyridine]dithiocyanatocobalt(II) (Form_I) Crystal data

**Table:**

[Co(NCS)_2_(C_7_H_10_N_2_)_2_]	*F*(000) = 434
*M**_r_* = 419.43	*D*_x_ = 1.462 Mg m^−^^3^
Monoclinic, *P*12_1_/*m*1	Cu *K*α radiation, λ = 1.54184 Å
*a* = 5.3708 (1) Å	Cell parameters from 13880 reflections
*b* = 15.2200 (2) Å	θ = 3.8–77.3°
*c* = 11.8014 (1) Å	µ = 9.20 mm^−^^1^
β = 99.076 (1)°	*T* = 100 K
*V* = 952.61 (2) Å^3^	Block, light blue
*Z* = 2	0.2 × 0.12 × 0.04 mm

### Bis[4-(dimethylamino)pyridine]dithiocyanatocobalt(II) (Form_I) Data collection

**Table:**

XtaLAB Synergy, Dualflex, HyPix diffractometer	2100 independent reflections
Radiation source: micro-focus sealed X-ray tube, PhotonJet (Cu) X-ray Source	2081 reflections with *I* > 2σ(*I*)
Mirror monochromator	*R*_int_ = 0.025
Detector resolution: 10.0000 pixels mm^-1^	θ_max_ = 78.1°, θ_min_ = 3.8°
ω scans	*h* = −6→5
Absorption correction: multi-scan (CrysalisPro; Rigaku OD, 2021)	*k* = −19→19
*T*_min_ = 0.311, *T*_max_ = 1.000	*l* = −14→14
16411 measured reflections	

### Bis[4-(dimethylamino)pyridine]dithiocyanatocobalt(II) (Form_I) Refinement

**Table:**

Refinement on *F*^2^	Primary atom site location: dual
Least-squares matrix: full	Hydrogen site location: inferred from neighbouring sites
*R*[*F*^2^ > 2σ(*F*^2^)] = 0.024	H-atom parameters constrained
*wR*(*F*^2^) = 0.067	*w* = 1/[σ^2^(*F*_o_^2^) + (0.0348*P*)^2^ + 0.4033*P*] where *P* = (*F*_o_^2^ + 2*F*_c_^2^)/3
*S* = 1.14	(Δ/σ)_max_ = 0.001
2100 reflections	Δρ_max_ = 0.25 e Å^−^^3^
126 parameters	Δρ_min_ = −0.37 e Å^−^^3^
0 restraints	

### Bis[4-(dimethylamino)pyridine]dithiocyanatocobalt(II) (Form_I) Special details

**Table:**

Geometry. All esds (except the esd in the dihedral angle between two l.s. planes) are estimated using the full covariance matrix. The cell esds are taken into account individually in the estimation of esds in distances, angles and torsion angles; correlations between esds in cell parameters are only used when they are defined by crystal symmetry. An approximate (isotropic) treatment of cell esds is used for estimating esds involving l.s. planes.

### Bis[4-(dimethylamino)pyridine]dithiocyanatocobalt(II) (Form_I) Fractional atomic coordinates and isotropic or equivalent isotropic displacement parameters (Å^2^)

**Table:**

	*x*	*y*	*z*	*U*_iso_*/*U*_eq_	
Co1	0.93113 (6)	0.750000	0.27609 (3)	0.01820 (10)	
N1	1.1349 (3)	0.750000	0.15392 (15)	0.0236 (4)	
C1	1.2586 (4)	0.750000	0.08142 (17)	0.0197 (4)	
S1	1.43347 (10)	0.750000	−0.01905 (4)	0.02516 (13)	
N2	1.1055 (3)	0.750000	0.43574 (15)	0.0228 (4)	
C2	1.2525 (4)	0.750000	0.52018 (17)	0.0202 (4)	
S2	1.46184 (10)	0.750000	0.63528 (4)	0.02618 (13)	
N11	0.7113 (2)	0.64220 (8)	0.26788 (10)	0.0186 (2)	
C11	0.5802 (3)	0.62788 (9)	0.35490 (12)	0.0194 (3)	
H11	0.614076	0.664879	0.420344	0.023*	
C12	0.4021 (3)	0.56372 (9)	0.35509 (12)	0.0197 (3)	
H12	0.318372	0.556780	0.419671	0.024*	
C13	0.3428 (3)	0.50769 (9)	0.25904 (12)	0.0196 (3)	
C14	0.4861 (3)	0.52099 (10)	0.16919 (12)	0.0216 (3)	
H14	0.460807	0.483952	0.103587	0.026*	
C15	0.6609 (3)	0.58727 (9)	0.17704 (12)	0.0203 (3)	
H15	0.752119	0.595055	0.115020	0.024*	
N12	0.1612 (2)	0.44622 (8)	0.25328 (11)	0.0229 (3)	
C16	0.0279 (3)	0.43142 (10)	0.34987 (14)	0.0265 (3)	
H16A	0.147210	0.410913	0.416077	0.040*	
H16B	−0.103220	0.386937	0.329058	0.040*	
H16C	−0.049866	0.486459	0.369491	0.040*	
C17	0.1036 (3)	0.38967 (11)	0.15329 (15)	0.0320 (4)	
H17A	0.053675	0.425800	0.084731	0.048*	
H17B	−0.034880	0.349998	0.163508	0.048*	
H17C	0.253102	0.355095	0.144230	0.048*	

### Bis[4-(dimethylamino)pyridine]dithiocyanatocobalt(II) (Form_I) Atomic displacement parameters (Å^2^)

**Table:**

	*U*^11^	*U*^22^	*U*^33^	*U*^12^	*U*^13^	*U*^23^
Co1	0.01752 (17)	0.01727 (17)	0.02091 (17)	0.000	0.00643 (12)	0.000
N1	0.0219 (8)	0.0256 (9)	0.0246 (9)	0.000	0.0077 (7)	0.000
C1	0.0170 (9)	0.0193 (9)	0.0220 (9)	0.000	0.0007 (7)	0.000
S1	0.0251 (3)	0.0307 (3)	0.0215 (2)	0.000	0.00917 (19)	0.000
N2	0.0225 (8)	0.0226 (9)	0.0246 (9)	0.000	0.0076 (7)	0.000
C2	0.0223 (9)	0.0160 (9)	0.0248 (10)	0.000	0.0108 (8)	0.000
S2	0.0249 (3)	0.0292 (3)	0.0237 (2)	0.000	0.00158 (19)	0.000
N11	0.0187 (5)	0.0172 (5)	0.0203 (5)	0.0012 (4)	0.0043 (4)	0.0012 (4)
C11	0.0211 (6)	0.0187 (6)	0.0190 (6)	0.0022 (5)	0.0047 (5)	−0.0010 (5)
C12	0.0218 (6)	0.0195 (7)	0.0191 (6)	0.0030 (5)	0.0071 (5)	0.0015 (5)
C13	0.0179 (6)	0.0179 (6)	0.0229 (6)	0.0021 (5)	0.0027 (5)	0.0015 (5)
C14	0.0242 (7)	0.0221 (7)	0.0187 (6)	0.0011 (6)	0.0041 (5)	−0.0030 (5)
C15	0.0220 (6)	0.0217 (7)	0.0181 (6)	0.0023 (6)	0.0062 (5)	0.0003 (5)
N12	0.0222 (6)	0.0196 (6)	0.0275 (6)	−0.0022 (5)	0.0055 (5)	−0.0016 (5)
C16	0.0234 (7)	0.0246 (7)	0.0324 (8)	−0.0020 (6)	0.0067 (6)	0.0059 (6)
C17	0.0302 (8)	0.0280 (8)	0.0379 (9)	−0.0072 (7)	0.0056 (7)	−0.0099 (7)

### Bis[4-(dimethylamino)pyridine]dithiocyanatocobalt(II) (Form_I) Geometric parameters (Å, º)

**Table:**

Co1—N1	1.9429 (18)	C13—C14	1.4195 (19)
Co1—N2	1.9672 (19)	C13—N12	1.3454 (19)
Co1—N11	2.0148 (12)	C14—H14	0.9500
Co1—N11^i^	2.0148 (12)	C14—C15	1.371 (2)
N1—C1	1.163 (3)	C15—H15	0.9500
C1—S1	1.625 (2)	N12—C16	1.4559 (19)
N2—C2	1.170 (3)	N12—C17	1.454 (2)
C2—S2	1.621 (2)	C16—H16A	0.9800
N11—C11	1.3512 (17)	C16—H16B	0.9800
N11—C15	1.3529 (18)	C16—H16C	0.9800
C11—H11	0.9500	C17—H17A	0.9800
C11—C12	1.367 (2)	C17—H17B	0.9800
C12—H12	0.9500	C17—H17C	0.9800
C12—C13	1.414 (2)		
			
N1—Co1—N2	118.16 (8)	C13—C14—H14	120.0
N1—Co1—N11	111.03 (4)	C15—C14—C13	120.05 (13)
N1—Co1—N11^i^	111.03 (4)	C15—C14—H14	120.0
N2—Co1—N11	103.47 (4)	N11—C15—C14	123.86 (13)
N2—Co1—N11^i^	103.47 (4)	N11—C15—H15	118.1
N11—Co1—N11^i^	109.04 (7)	C14—C15—H15	118.1
C1—N1—Co1	179.48 (17)	C13—N12—C16	120.66 (12)
N1—C1—S1	179.51 (19)	C13—N12—C17	120.83 (13)
C2—N2—Co1	166.24 (16)	C17—N12—C16	118.42 (12)
N2—C2—S2	178.57 (18)	N12—C16—H16A	109.5
C11—N11—Co1	117.81 (9)	N12—C16—H16B	109.5
C11—N11—C15	116.08 (12)	N12—C16—H16C	109.5
C15—N11—Co1	125.71 (9)	H16A—C16—H16B	109.5
N11—C11—H11	117.8	H16A—C16—H16C	109.5
N11—C11—C12	124.41 (13)	H16B—C16—H16C	109.5
C12—C11—H11	117.8	N12—C17—H17A	109.5
C11—C12—H12	120.0	N12—C17—H17B	109.5
C11—C12—C13	119.91 (13)	N12—C17—H17C	109.5
C13—C12—H12	120.0	H17A—C17—H17B	109.5
C12—C13—C14	115.63 (13)	H17A—C17—H17C	109.5
N12—C13—C12	121.99 (13)	H17B—C17—H17C	109.5
N12—C13—C14	122.38 (13)		

Symmetry code: (i) *x*, −*y*+3/2, *z*.

### Bis[4-(dimethylamino)pyridine]dithiocyanatocobalt(II) (Form_I) Hydrogen-bond geometry (Å, º)

**Table:**

*D*—H···*A*	*D*—H	H···*A*	*D*···*A*	*D*—H···*A*
C16—H16*B*···S2^ii^	0.98	2.91	3.8367 (15)	158
C17—H17*B*···S1^iii^	0.98	2.94	3.7291 (16)	138
C17—H17*C*···S1^iv^	0.98	2.89	3.8018 (18)	155

Symmetry codes: (ii) −*x*+1, −*y*+1, −*z*+1; (iii) −*x*+1, −*y*+1, −*z*; (iv) −*x*+2, −*y*+1, −*z*.

### (Form_II) Crystal data

**Table:**

C_16_H_20_CoN_6_S_2_	*F*(000) = 868
*M**_r_* = 419.43	*D*_x_ = 1.457 Mg m^−^^3^
Monoclinic, *P*2_1_/*c*	Cu *K*α radiation, λ = 1.54184 Å
*a* = 13.9171 (1) Å	Cell parameters from 41675 reflections
*b* = 9.5114 (1) Å	θ = 3.2–79.4°
*c* = 14.4487 (1) Å	µ = 9.17 mm^−^^1^
β = 90.489 (1)°	*T* = 100 K
*V* = 1912.52 (3) Å^3^	Block, dark blue
*Z* = 4	0.18 × 0.1 × 0.03 mm

### (Form_II) Data collection

**Table:**

XtaLAB Synergy, Dualflex, HyPix diffractometer	4151 independent reflections
Radiation source: micro-focus sealed X-ray tube, PhotonJet (Cu) X-ray Source	4079 reflections with *I* > 2σ(*I*)
Mirror monochromator	*R*_int_ = 0.025
Detector resolution: 10.0000 pixels mm^-1^	θ_max_ = 80.2°, θ_min_ = 3.2°
ω scans	*h* = −17→17
Absorption correction: multi-scan (CrysalisPro; Rigaku OD, 2021)	*k* = −12→12
*T*_min_ = 0.476, *T*_max_ = 1.000	*l* = −18→18
57319 measured reflections	

### (Form_II) Refinement

**Table:**

Refinement on *F*^2^	Hydrogen site location: inferred from neighbouring sites
Least-squares matrix: full	H-atom parameters constrained
*R*[*F*^2^ > 2σ(*F*^2^)] = 0.026	*w* = 1/[σ^2^(*F*_o_^2^) + (0.0406*P*)^2^ + 0.8179*P*] where *P* = (*F*_o_^2^ + 2*F*_c_^2^)/3
*wR*(*F*^2^) = 0.073	(Δ/σ)_max_ = 0.002
*S* = 1.13	Δρ_max_ = 0.29 e Å^−^^3^
4151 reflections	Δρ_min_ = −0.35 e Å^−^^3^
231 parameters	Extinction correction: *SHELXL2016/6* (Sheldrick, 2015b), Fc^*^=kFc[1+0.001xFc^2^λ^3^/sin(2θ)]^-1/4^
0 restraints	Extinction coefficient: 0.00076 (11)
Primary atom site location: dual	

### (Form_II) Special details

**Table:**

Geometry. All esds (except the esd in the dihedral angle between two l.s. planes) are estimated using the full covariance matrix. The cell esds are taken into account individually in the estimation of esds in distances, angles and torsion angles; correlations between esds in cell parameters are only used when they are defined by crystal symmetry. An approximate (isotropic) treatment of cell esds is used for estimating esds involving l.s. planes.

### (Form_II) Fractional atomic coordinates and isotropic or equivalent isotropic displacement parameters (Å^2^)

**Table:**

	*x*	*y*	*z*	*U*_iso_*/*U*_eq_	
Co1	0.77328 (2)	0.36737 (2)	0.79003 (2)	0.01802 (8)	
N1	0.89733 (9)	0.40269 (14)	0.84966 (9)	0.0228 (3)	
C1	0.97734 (11)	0.42519 (15)	0.87028 (9)	0.0203 (3)	
S1	1.08865 (3)	0.45439 (4)	0.89675 (3)	0.02797 (10)	
N2	0.72639 (9)	0.17383 (14)	0.78687 (9)	0.0249 (3)	
C2	0.69467 (10)	0.06052 (16)	0.79023 (10)	0.0225 (3)	
S2	0.64780 (3)	−0.09564 (4)	0.79675 (3)	0.02997 (10)	
N11	0.67807 (8)	0.48667 (13)	0.85774 (8)	0.0203 (2)	
C11	0.69896 (10)	0.53200 (16)	0.94415 (10)	0.0213 (3)	
H11	0.759338	0.506648	0.970392	0.026*	
C12	0.63811 (11)	0.61250 (16)	0.99628 (10)	0.0218 (3)	
H12	0.656100	0.639410	1.057333	0.026*	
C13	0.54861 (10)	0.65531 (15)	0.95897 (10)	0.0199 (3)	
C14	0.52571 (10)	0.60389 (16)	0.86934 (10)	0.0222 (3)	
H14	0.465517	0.625731	0.841373	0.027*	
C15	0.59106 (10)	0.52243 (16)	0.82332 (10)	0.0213 (3)	
H15	0.573977	0.489080	0.763427	0.026*	
N12	0.48862 (9)	0.74021 (14)	1.00607 (9)	0.0241 (3)	
C16	0.51936 (12)	0.80309 (19)	1.09350 (12)	0.0303 (3)	
H16A	0.577275	0.859533	1.083572	0.045*	
H16B	0.533416	0.728603	1.138480	0.045*	
H16C	0.468041	0.863388	1.117198	0.045*	
C17	0.40047 (11)	0.79162 (19)	0.96272 (12)	0.0309 (3)	
H17A	0.360713	0.711636	0.943368	0.046*	
H17B	0.416451	0.848679	0.908512	0.046*	
H17C	0.364953	0.849121	1.007120	0.046*	
N21	0.78919 (8)	0.42769 (13)	0.65835 (8)	0.0189 (2)	
C21	0.82983 (10)	0.33442 (16)	0.59926 (10)	0.0196 (3)	
H21	0.834119	0.238949	0.618167	0.024*	
C22	0.86504 (11)	0.36954 (15)	0.51404 (10)	0.0209 (3)	
H22	0.891632	0.299061	0.475335	0.025*	
C23	0.86172 (10)	0.51100 (16)	0.48379 (9)	0.0200 (3)	
C24	0.81437 (11)	0.60652 (16)	0.54359 (11)	0.0227 (3)	
H24	0.805027	0.701424	0.525107	0.027*	
C25	0.78214 (10)	0.56180 (15)	0.62799 (10)	0.0211 (3)	
H25	0.753116	0.629049	0.667518	0.025*	
N22	0.90343 (9)	0.55389 (14)	0.40487 (8)	0.0231 (3)	
C26	0.95644 (11)	0.45537 (17)	0.34714 (10)	0.0248 (3)	
H26A	0.911620	0.406459	0.305536	0.037*	
H26B	0.989513	0.386597	0.386600	0.037*	
H26C	1.003783	0.506881	0.310500	0.037*	
C27	0.89616 (12)	0.69988 (18)	0.37410 (11)	0.0290 (3)	
H27A	0.927850	0.761294	0.419469	0.043*	
H27B	0.828308	0.726211	0.368232	0.043*	
H27C	0.927463	0.710043	0.313971	0.043*	

### (Form_II) Atomic displacement parameters (Å^2^)

**Table:**

	*U*^11^	*U*^22^	*U*^33^	*U*^12^	*U*^13^	*U*^23^
Co1	0.01610 (13)	0.02028 (13)	0.01770 (13)	−0.00148 (8)	0.00179 (9)	−0.00024 (8)
N1	0.0219 (6)	0.0263 (6)	0.0202 (6)	−0.0016 (5)	0.0008 (5)	0.0020 (5)
C1	0.0247 (7)	0.0197 (7)	0.0166 (6)	−0.0006 (5)	0.0008 (5)	0.0013 (5)
S1	0.02121 (18)	0.0317 (2)	0.03093 (19)	−0.00319 (14)	−0.00599 (14)	0.00401 (15)
N2	0.0239 (6)	0.0224 (6)	0.0284 (6)	−0.0016 (5)	0.0022 (5)	0.0000 (5)
C2	0.0175 (6)	0.0262 (8)	0.0238 (7)	0.0019 (6)	−0.0009 (5)	0.0005 (6)
S2	0.02535 (19)	0.02148 (19)	0.0430 (2)	−0.00320 (14)	−0.00451 (16)	0.00357 (16)
N11	0.0187 (6)	0.0227 (6)	0.0196 (5)	−0.0013 (5)	0.0015 (4)	−0.0004 (5)
C11	0.0166 (6)	0.0262 (7)	0.0211 (6)	−0.0004 (5)	−0.0011 (5)	−0.0012 (6)
C12	0.0195 (7)	0.0248 (7)	0.0210 (7)	−0.0013 (5)	−0.0012 (5)	−0.0016 (5)
C13	0.0170 (6)	0.0193 (6)	0.0235 (7)	−0.0026 (5)	0.0022 (5)	0.0020 (5)
C14	0.0170 (6)	0.0259 (7)	0.0236 (7)	−0.0009 (5)	−0.0034 (5)	0.0031 (6)
C15	0.0210 (7)	0.0243 (7)	0.0187 (6)	−0.0019 (5)	−0.0015 (5)	0.0002 (5)
N12	0.0187 (6)	0.0264 (6)	0.0270 (6)	0.0034 (5)	0.0012 (5)	−0.0015 (5)
C16	0.0245 (7)	0.0333 (9)	0.0332 (8)	0.0030 (6)	0.0030 (6)	−0.0107 (7)
C17	0.0224 (7)	0.0324 (8)	0.0378 (8)	0.0089 (6)	−0.0004 (6)	0.0007 (7)
N21	0.0170 (5)	0.0212 (6)	0.0185 (5)	−0.0003 (4)	0.0010 (4)	−0.0005 (4)
C21	0.0188 (6)	0.0193 (6)	0.0207 (6)	−0.0009 (5)	0.0007 (5)	−0.0015 (5)
C22	0.0183 (7)	0.0241 (7)	0.0203 (7)	−0.0007 (5)	0.0003 (5)	−0.0034 (5)
C23	0.0153 (6)	0.0253 (7)	0.0194 (6)	−0.0031 (5)	−0.0016 (5)	−0.0005 (5)
C24	0.0215 (7)	0.0210 (7)	0.0257 (7)	0.0010 (5)	0.0004 (6)	0.0025 (6)
C25	0.0195 (6)	0.0208 (7)	0.0228 (7)	0.0024 (5)	0.0014 (5)	−0.0022 (5)
N22	0.0226 (6)	0.0262 (7)	0.0205 (6)	−0.0024 (5)	0.0029 (5)	0.0018 (5)
C26	0.0216 (7)	0.0333 (8)	0.0195 (7)	−0.0043 (6)	0.0037 (5)	−0.0005 (6)
C27	0.0308 (8)	0.0293 (8)	0.0269 (7)	−0.0040 (6)	0.0021 (6)	0.0076 (6)

### (Form_II) Geometric parameters (Å, º)

**Table:**

Co1—N1	1.9521 (13)	C16—H16C	0.9800
Co1—N2	1.9535 (14)	C17—H17A	0.9800
Co1—N11	2.0057 (12)	C17—H17B	0.9800
Co1—N21	2.0013 (12)	C17—H17C	0.9800
N1—C1	1.170 (2)	N21—C21	1.3579 (18)
C1—S1	1.6164 (15)	N21—C25	1.3522 (19)
N2—C2	1.166 (2)	C21—H21	0.9500
C2—S2	1.6253 (16)	C21—C22	1.371 (2)
N11—C11	1.3502 (18)	C22—H22	0.9500
N11—C15	1.3488 (19)	C22—C23	1.415 (2)
C11—H11	0.9500	C23—C24	1.420 (2)
C11—C12	1.372 (2)	C23—N22	1.3472 (18)
C12—H12	0.9500	C24—H24	0.9500
C12—C13	1.413 (2)	C24—C25	1.370 (2)
C13—C14	1.418 (2)	C25—H25	0.9500
C13—N12	1.3498 (19)	N22—C26	1.4590 (19)
C14—H14	0.9500	N22—C27	1.461 (2)
C14—C15	1.371 (2)	C26—H26A	0.9800
C15—H15	0.9500	C26—H26B	0.9800
N12—C16	1.459 (2)	C26—H26C	0.9800
N12—C17	1.457 (2)	C27—H27A	0.9800
C16—H16A	0.9800	C27—H27B	0.9800
C16—H16B	0.9800	C27—H27C	0.9800
			
N1—Co1—N2	117.81 (6)	N12—C17—H17B	109.5
N1—Co1—N11	105.83 (5)	N12—C17—H17C	109.5
N1—Co1—N21	105.41 (5)	H17A—C17—H17B	109.5
N2—Co1—N11	108.82 (5)	H17A—C17—H17C	109.5
N2—Co1—N21	106.71 (5)	H17B—C17—H17C	109.5
N21—Co1—N11	112.36 (5)	C21—N21—Co1	117.41 (10)
C1—N1—Co1	168.56 (12)	C25—N21—Co1	124.79 (10)
N1—C1—S1	178.72 (14)	C25—N21—C21	116.22 (12)
C2—N2—Co1	175.34 (13)	N21—C21—H21	118.0
N2—C2—S2	178.29 (15)	N21—C21—C22	124.03 (14)
C11—N11—Co1	119.59 (10)	C22—C21—H21	118.0
C15—N11—Co1	123.89 (10)	C21—C22—H22	120.1
C15—N11—C11	116.49 (12)	C21—C22—C23	119.87 (13)
N11—C11—H11	118.1	C23—C22—H22	120.1
N11—C11—C12	123.84 (13)	C22—C23—C24	115.75 (13)
C12—C11—H11	118.1	N22—C23—C22	122.41 (13)
C11—C12—H12	120.1	N22—C23—C24	121.80 (14)
C11—C12—C13	119.85 (14)	C23—C24—H24	120.0
C13—C12—H12	120.1	C25—C24—C23	120.00 (14)
C12—C13—C14	116.08 (13)	C25—C24—H24	120.0
N12—C13—C12	121.80 (14)	N21—C25—C24	123.92 (13)
N12—C13—C14	122.12 (14)	N21—C25—H25	118.0
C13—C14—H14	120.2	C24—C25—H25	118.0
C15—C14—C13	119.54 (13)	C23—N22—C26	120.87 (13)
C15—C14—H14	120.2	C23—N22—C27	121.07 (13)
N11—C15—C14	124.11 (13)	C26—N22—C27	118.06 (12)
N11—C15—H15	117.9	N22—C26—H26A	109.5
C14—C15—H15	117.9	N22—C26—H26B	109.5
C13—N12—C16	120.26 (13)	N22—C26—H26C	109.5
C13—N12—C17	120.40 (13)	H26A—C26—H26B	109.5
C17—N12—C16	118.32 (13)	H26A—C26—H26C	109.5
N12—C16—H16A	109.5	H26B—C26—H26C	109.5
N12—C16—H16B	109.5	N22—C27—H27A	109.5
N12—C16—H16C	109.5	N22—C27—H27B	109.5
H16A—C16—H16B	109.5	N22—C27—H27C	109.5
H16A—C16—H16C	109.5	H27A—C27—H27B	109.5
H16B—C16—H16C	109.5	H27A—C27—H27C	109.5
N12—C17—H17A	109.5	H27B—C27—H27C	109.5

### (Form_II) Hydrogen-bond geometry (Å, º)

**Table:**

*D*—H···*A*	*D*—H	H···*A*	*D*···*A*	*D*—H···*A*
C11—H11···S1^i^	0.95	2.87	3.7312 (15)	152
C16—H16*C*···S2^ii^	0.98	3.01	3.9663 (16)	166
C21—H21···S1^iii^	0.95	2.92	3.7888 (15)	153
C22—H22···N1^iv^	0.95	2.64	3.5448 (19)	159
C26—H26*A*···N2^iv^	0.98	2.70	3.531 (2)	143
C27—H27*B*···S2^iv^	0.98	2.98	3.7568 (17)	137

Symmetry codes: (i) −*x*+2, −*y*+1, −*z*+2; (ii) −*x*+1, −*y*+1, −*z*+2; (iii) −*x*+2, *y*−1/2, −*z*+3/2; (iv) *x*, −*y*+1/2, *z*−1/2.
